# Differential Patterns of Social Attention and Memory Profiles in Depression: Evidence From Third‐Person Social Interaction Processing

**DOI:** 10.1155/da/7518415

**Published:** 2026-06-30

**Authors:** Enze Tang, Xiao-Mei Zhang, Jian Li, Yueyao Liu, Zhong-Xia Shen, Hui Chen

**Affiliations:** ^1^ The First Affiliated Hospital, Zhejiang University School of Medicine, Hangzhou, China, zju.edu.cn; ^2^ Department of Psychology and Behavioral Sciences, Zhejiang University, Hangzhou, China, zju.edu.cn; ^3^ Department of Psychiatry, Brigham and Women’s Hospital, Harvard Medical School, Boston, Massachusetts, USA, harvard.edu; ^4^ Department of Psychiatry, Huzhou Third Municipal Hospital, Huzhou, China; ^5^ Nanjing Brain Hospital Affiliated to Nanjing Medical University, Nanjing, China, njmu.edu.cn; ^6^ Zhejiang Key Laboratory of Precision Psychiatry, Hangzhou, China

**Keywords:** attention, major depressive disorder, memory, social cognition, social interaction

## Abstract

Although social interaction is crucial for human social life, it poses unique challenges for individuals with depressive symptoms. From a third‐person perspective, this study employed two cross‐sectional experiments to investigate how depressive symptoms modulate whether the benefits of social interaction transfer to attention and memory functions, which constitute core processes underpinning successful interpersonal relationships and social engagement. A total of 72 patients with major depressive disorder (MDD) and 72 demographically matched healthy control subjects (HCS) were recruited. Experiment 1 adopted a visual search (VS) task in which participants identified socially interactive or noninteractive individuals amid distractors. Experiment 2 included a working memory (WM) task focusing on interpersonal spatial distance and a long‐term memory (LTM) task requiring the recollection of paired relationships. Compared to HCS, MDD patients exhibited preserved social attention benefits induced by social interactions, as evidenced by their higher search efficiency for socially interactive dyads. In contrast, MDD patients showed no evidence of either spatial compression effects induced by social intimacy in working memory or enhanced memory traces for interactive dyads in LTM. These findings suggest that individuals with depressive symptoms can benefit from social interactions to facilitate attention, but not memory functioning. Uncovering these differential patterns of attention and memory performance in social cognition challenges the traditional conceptualization of global social cognitive impairment in depression. The current findings also pave the way for targeted social memory training programs and interventions aimed at improving the quality of life in this population.

## 1. Introduction

Major depressive disorder (MDD) is a severe psychiatric disorder afflicting millions of people worldwide every year, and it has become the leading cause of disability in China [1]. Core symptoms of MDD include low mood and alterations in affective responsiveness [2], which can fundamentally reshape how socially relevant information is processed. Indeed, individuals with MDD often exhibit reduced sensitivity to cues that typically signal social relevance [3], and such changes in social cognition have been associated with the severity of depressive symptoms [4].

Social interactions represent a particularly important class of social information, as they convey relational structure and potential social meaning without relying on explicit emotional expressions or deliberate social judgments. In healthy individuals, configurations implying social interaction, as represented by two agents facing toward each other, typically receive preferential cognitive processing, including enhanced attentional efficiency [5] and strengthened memory representations [6]. These effects suggest that social interaction itself can function as an intrinsic organizing principle for cognition [7–10]. In the context of MDD, however, this intrinsic facilitation of social interaction may be disrupted. Depression is characterized by profound social impairments and interpersonal avoidance, which may attenuate the cognitive prioritization typically afforded to socially meaningful configurations [11–13]. As a result, socially interactive scenes may no longer elicit processing advantages in MDD, even when they are perceptually simple and devoid of explicit emotional content.

Importantly, previous research on social interaction in MDD has emphasized first‐person interpersonal experience [14], whereas relatively little attention has been paid to how socially structured information is processed from a third‐person perspective. In fact, many everyday social situations require individuals to extract relational information from observed interactions without immediate personal involvement, such as situations where we are newcomers to a community or a workplace. In MDD, the cognitive domains of social attention and memory are particularly vulnerable to affective modulation [15, 16], and social interaction cues may influence these domains differently by altering the perceptual and mnemonic weighting of relational information [17]. Examining how such cues are processed in attention and memory therefore provides a useful approach for characterizing the impact of depressive states on social cognition from a novel perspective.

In fact, the ability to process such third‐party interactions holds particular clinical relevance for understanding the social phenotypes of MDD, linking basic cognitive deficits to real‐world behavioral symptoms. First, depressive symptoms are associated with specific patterns of social engagement, which could be relevant to how these symptoms influence patients’ observation and understanding of social interactions among other people. Recent social network analyses have increasingly supported the “dyadic‐isolation hypothesis,” indicating that individuals with higher levels of depressive symptoms prefer interacting in isolated dyads rather than in larger social groups [18, 19]. However, the mechanisms underlying this depression‐related preference for smaller interaction groups remain poorly understood. One compelling possibility is that individuals with depression struggle to efficiently process the complex multi‐agent social cues embedded in others’ interactions [20]. By examining how MDD patients cognitively process fundamental third‐party interactions, our study provides a foundational building block. If patients fail to automatically prioritize and bind even the simplest social interactions as observers, they will inevitably be ill‐equipped to navigate or engage in complex group‐level social networks. Alternatively, if their basic cognitive processing of others’ social cues remains intact, their withdrawal would more likely stem from purely psychosocial factors. Second, observing interactions is deeply tied to the core affective symptoms of MDD, specifically, social anhedonia. As a core hallmark of MDD, social anhedonia is typically associated with reduced sensitivity to social rewards and distinct brain biomarkers [21]. Similarly, theoretical frameworks also propose that depression is etiologically rooted in distorted social expectations [22] and blunted reward processing [23]. For healthy observers, face‐to‐face interactions inherently signal interpersonal closeness and positive social expectations, serving as intrinsic social rewards [24, 25]. Consequently, due to a distorted sensitivity to rewarding social cues, MDD patients may not be able to cognitively prioritize and retain relational information from the observed interactive dyads. Together, investigating how depressive symptoms influence how patients observe others’ interactions could deepen our mechanistic understanding of depression‐related social engagement preferences and identify cognitive precursors to their real‐life social withdrawal and interpersonal avoidance.

Despite the valuable insights provided by existing literature on social cognition in MDD, the current study provides a necessary and meaningful extension in several ways. The central research topics of previous research have heavily focused on classical theory‐of‐mind [26], self‐referential processing [27], and emotion processing, including emotion differentiation [28], facial emotion recognition [29], and affective prosody [30]. Converging evidence has revealed small‐to‐medium deficits in these social cognitive abilities, accompanied by distinct phenomena such as negative cognitive biases [31]. However, traditional paradigms primarily require patients to make explicit and deliberate interpretations of single social cues, leaving largely unknown how MDD patients process the implicit multi‐agent relationships embodied by abstract configurations. Such a paradigm shift is necessary to capture the pre‐reflective cognitive biases that drive spontaneous real‐world social withdrawal [32]. Second, previous studies have predominantly focused on the first‐person processing of social stimuli (e.g., how a patient directly relates to a social target), but real‐world social environments are fundamentally complex networks where individuals frequently act as third‐party observers. Mapping these allocentric relational structures is a necessary prerequisite for navigating social hierarchies and identifying social support systems [33], and its clinical importance has been increasingly recognized [34–36]. Therefore, investigating this allocentric cognitive processing is a necessary extension to uncover a critical but previously missing foundational mechanism underlying key social phenotypes in MDD, such as social isolation. Finally, the majority of existing literature measures the ability to evaluate the social stimulus itself, without examining how this processing interacts with broader cognitive domains. Simply knowing whether a patient can recognize a stimulus does not explain why they fail to engage with it in daily life. Contemporary models of motivational deficits in MDD highlight that the core issue is often not a perceptual inability but a failure to assign value to social cues, preventing them from being prioritized in attention and memory [37]. To address this, the current study extends the scope by investigating whether embedded social information benefits fundamental attention and memory performance, revealing whether MDD patients retain a cognitive prioritization for socially interactive stimuli.

The present study aimed to investigate how individuals with MDD process socially interactive versus noninteractive dyadic configurations in the attention (Experiment 1) and memory (Experiment 2) domains. Experiment 1 assessed attentional processing using a visual search (VS) paradigm [5, 34] to determine whether social interaction cues facilitate search efficiency in MDD. Experiment 2 examined memory‐related processing through a spatial working memory (WM) task and a surprise long‐term memory (LTM) task [6, 35], probing whether social interaction enhances mnemonic representations. Across both experiments, social interaction was manipulated by the relative orientation of paired agents (face‐to‐face vs. back‐to‐back), allowing for the examination of the cognitive impact of social relational structures while minimizing task‐irrelevant demands or confounds. We hypothesized that, compared to healthy controls, individuals with MDD would show attenuated or absent facilitation effects of social interaction across attention and memory domains.

## 2. Methods

### 2.1. Participants

Prior power analyses were conducted using G^∗^Power 3.1.9 [38] to determine the minimum sample size required for detecting medium‐sized effects in the planned mixed‐design analyses of variance (ANOVA). Based on previous studies employing similar experimental paradigms [34, 36], a minimum of 34 participants was required to achieve 80% statistical power with an alpha level of 0.05. Therefore, independent samples of 36 patients with MDD and 36 healthy control subjects (HCS) were recruited for Experiment 1 and Experiment 2, respectively.

Data collection was carried out between July 2024 and April 2025. Participants with MDD were recruited from Huzhou Third Municipal Hospital in China. Inclusion criteria for the MDD group were as follows: (1) age between 18 and 60 years; (2) right‐handedness; (3) a current diagnosis of MDD confirmed independently by two psychiatrists according to the International Classification of Diseases, 10th revision (ICD‐10; [39]); and (4) a total score of at least 17 on the 17‐item Hamilton Depression Rating Scale (HAMD‐17; [40]). All participants were receiving stable pharmacological treatment during participation. The medication regimens included selective serotonin reuptake inhibitors (SSRIs; Experiment 1: 58.3%; Experiment 2: 66.7%), serotonin and norepinephrine reuptake inhibitors (SNRIs; 25.0%; 19.4%) or combined treatment (16.7%; 13.9%). As for treatment history, a proportion of patients had received at least one prior course of antidepressant treatment (Experiment 1: 80.6%; Experiment 2: 83.3%), noninvasive brain stimulation treatment (47.2%; 52.8%), as well as cognitive behavioral therapy or supportive psychotherapy (27.8%; 22.2%). No patients were receiving psychotherapy during participation. HCS were recruited through community advertisements and screened using the Mini‐International Neuropsychiatric Interview (M.I.N.I.; [41]) to confirm the absence of any current or past psychiatric disorders. General exclusion criteria for all participants included: (1) comorbid psychiatric diagnoses; (2) transcranial magnetic stimulation or electroconvulsive therapy within the past month; (3) a history of severe head injury, loss of consciousness for more than 5 min, or any neurological disorders; (4) a history of alcohol or substance dependence or abuse; and (5) higher than six points on the Young Mania Rating Scale (YMRS; [42]).

As shown in Table 1, the MDD and HCS groups were matched for age, sex distribution, and education in both experiments. Clinical symptom severity was assessed using the Patient Health Questionnaire‐9 (PHQ‐9; [43]), the Beck Depression Inventory Second Edition (BDI‐II; [44]), the State‐Trait Anxiety Inventory (STAI; [45]), and the 20‐item brief version of the Positive and Negative Affect Schedule (PANAS; [46]). Although the HCS samples exhibited nontrivial scores on the PHQ‐9 and BDI‐II scales, these values fell strictly within the established ranges for nonclinical general populations [47, 48] and were significantly lower than the corresponding MDD samples, reflecting normal mood fluctuations rather than clinical pathology. The study protocol was approved by the Institutional Review Board of the Department of Psychology and Behavioral Sciences, Zhejiang University (Approval No. 2022087). Written informed consent was obtained from all participants prior to participation.

**Table 1:** Demographic characteristics of participants.

Items	Experiment 1			Experiment 2		
	MDD (*N* = 36)	HCS (*n* = 36)	Statistics	MDD (*N* = 36)	HCS (*n* = 36)	Statistics
Age (years)	41.67 (12.07)	38.75 (7.53)	*t* (70) = 1.23, *p* = 0.22	42.31 (12.01)	40.44 (8.02)	*t* (70) = 0.77, *p* = 0.44
Sex (M:F)	11:25	16:20	*χ* ^2^ = 1.48, *p* = 0.22	10:26	14:22	*χ* ^2^ = 1.00, *p* = 0.32
Education (years)	10.86 (3.41)	12.08 (3.10)	*t* (70) = 1.59, *p* = 0.12	10.94 (3.71)	11.22 (4.92)	*t* (70) = 0.27, *p* = 0.79
Illness duration (months)	52.47 (33.32)			58.94 (36.98)		
Episode number	2.36 (1.07)			2.53 (1.21)		
Fluoxetine equivalent (mg)	28.06 (10.37)			29.72 (9.41)		
PANAS‐P	23.33 (6.18)	30.06 (6.13)	*t* (70) = 4.63, *p* < 0.01	23.53 (6.00)	30.44 (6.01)	*t* (70) = 4.89, *p* < 0.01
PANAS‐N	24.06 (8.76)	23.17 (6.35)	*t* (70) = 0.49, *p* = 0.62	24.67 (8.09)	22.56 (6.07)	*t* (70) = 1.25, *p* = 0.22
PHQ‐9	11.22 (6.22)	6.72 (4.20)	*t* (70) = 3.60, *p* < 0.01	11.42 (5.81)	6.11 (4.03)	*t* (70) = 4.50, *p* < 0.01
BDI‐II	15.19 (12.52)	8.72 (8.85)	*t* (70) = 2.53, *p* = 0.01	15.25 (11.27)	8.06 (6.27)	*t* (70) = 3.35, *p* < 0.01
STAI‐S	48.72 (13.11)	40.67 (14.39)	*t* (70) = 2.48, *p* = 0.02	47.83 (12.53)	39.11 (13.31)	*t* (70) = 2.86, *p* < 0.01
STAI‐T	49.81 (14.29)	41.39 (13.43)	*t* (70) = 2.58, *p* = 0.01	50.00 (13.07)	40.17 (12.41)	*t* (70) = 3.27, *p* < 0.01
HAMD	20.00 (3.51)			20.42 (3.10)		
YMRS	2.48 (2.06)			2.72 (2.31)		

*Note:* Data are reported by mean (s.d.).

Abbreviations: BDI‐II, beck depression inventory second edition; HAMD, Hamilton depression scale; HCS, healthy control subjects; MDD, major depressive disorder; PANAS, positive and negative affect scale (positive or negative subscales); PHQ‐9, 9‐item patient health questionnaire; STAI, state‐trait anxiety inventory (state or trait subscales); YMRS, young manic rating scale.

### 2.2. Apparatus and Stimuli

Experiments were programed and executed by MATLAB software (The MathWorks; Natick, MA) with the Psychophysics Toolbox extension [49, 50]. All stimuli were displayed on a 15.6‐inch LED monitor, featuring a 60 Hz refresh rate and a 1920 × 1080 resolution. Throughout the experiment, participants maintained a viewing distance of ~50 cm.

**Figure 1:**
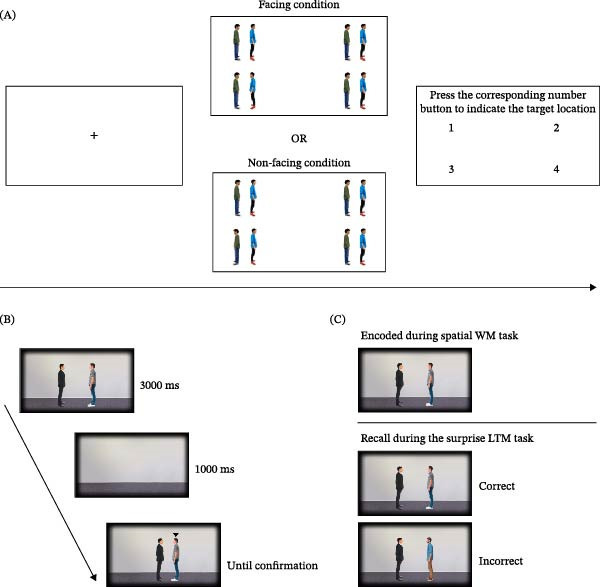
Experiment tasks and procedure. (A) Visual search task in Experiment 1. Participants searched for the block‐wise predetermined target (i.e., facing or non‐facing dyads) among distractors, and press the space bar immediately on seeing the target. Then, they indicated the location of the target by pressing the corresponding number key. (B) Working memory task in Experiment 2. Participants memorized the spatial distance between two models (either facing or non‐facing), and then moved the model indicated by a triangle leftwards or rightwards by pressing the arrow keys to restore the memorized between‐model distance as accurately as possible. (C) Long‐term memory task in Experiment 2. Participants tried to recall the pairing relationships of the models displayed in the working memory task.

For Experiment 1, pictures of two side‐view male models were sourced from the Adobe Stock Service, and both models had a neutral bodily expression in an upright standing gesture. The use of male‐only dyads was consistent with previous studies [6, 34], which mitigated the potential confounding effects of implicit higher‐order social semantics on the attentional prioritization driven by the facing configurations. Then, both left‐facing and right‐facing versions of each model were generated, which were further standardized to 350 pixels in height and 70 pixels in width. As shown in Figure 1A, four model pairs were presented together during the VS, where three pairs consisted of two models facing the same direction (i.e., distractors) and one pair (i.e., the target) was made up of either face‐to‐face dyads in the facing condition or back‐to‐back dyads in the non‐facing condition, with a between‐model distance of 140 pixels.

For Experiment 2, the stimuli comprised images of 20 male and 20 female models standing upright with neutral bodily expressions. All images were standardized to a visual angle of 4.77° × 23.45°. Individual images were combined to form 20 same‐sex pairs. For each participant, five male pairs and five female pairs were randomly assigned to the facing condition, while the remaining pairs were assigned to the non‐facing condition. The assignment of orientations was counterbalanced. All stimuli were displayed against a uniform background depicting an empty room.

### 2.3. Procedure and Design

Participants with MDD completed the experiments in a hospital psychometric testing room, and HCS completed the tasks in laboratory rooms at Zhejiang University. Each participant completed only one experiment. Clinical assessments were administered immediately after task completion.

For Experiment 1, participants were provided with the VS target (i.e., face‐to‐face or back‐to‐back dyads) before the beginning of each block, and they were required to search for that target throughout the following block. As illustrated in Figure 1A, each trial began with a fixation cross (0.46° × 0.46°) on the center of the screen, with a random duration of 500–1500 ms. Afterwards, the VS task was introduced with one target stimulus and three distractor stimuli presented at four quadrants of the screen. The quadrant wherein the target appeared was randomized on each trial. Participants were asked to search for the predetermined target as quickly as possible, and they should press the space bar immediately when they found the target. Then, numbers 1–4 were displayed on the screen to represent the four locations, and participants should accurately indicate the location of the target by pressing the corresponding number key. The next trial began after a 500 ms inter‐trial blank interval. Participants completed at least 6 practice trials for each condition before the formal experiment, and then completed 80 trials in Experiment 1 in two blocks of 40 trials (i.e., facing and non‐facing blocks). The order of blocks was counterbalanced across participants. Experiment 1 took about 10 min.

Experiment 2 consisted of a spatial WM task followed immediately by a surprise LTM task [6, 35]. In the WM task (Figure 1B), each trial began with the presentation of a background image for 500 ms, followed by the display of a pair of models for 3000 ms. During the encoding period, the initial memorized distance between the two models was not fixed, and the between‐model distance was randomly jittered on a trial‐by‐trial basis within a range of 300–480 pixels. Participants were instructed to memorize the between‐model distance. After a 1000 ms blank interval, the models reappeared at new locations with a different between‐model distance. To determine the new locations for the models’ reappearance, randomized numbers were generated to represent the central points of the paired stimuli, and the models were never displayed within the 20% horizontal pixel range to either side of the screen. For the distance reproduction display, the between‐model distance was never identical to the memorized distance, which was altered by randomly adding a distance change of −150, −100, 100, or +150 pixels. One model was indicated by an arrow cue above the head, and participants adjusted its horizontal position using the left or right arrow keys to reproduce the previously memorized distance. In this procedure, the other model without an arrow cue was spatially fixed on the screen, so that any change in distance was entirely induced by the movable model through key pressing. Each key press shifted the designated model by a fixed spatial increment of 10 pixels. The key press was collected discretely, waiting for the key release before registering the next move, which required participants to make successive and deliberate key presses rather than simply holding down the key to create a continuous and fast sliding motion. The reproduction phase was entirely self‐paced without time limit. Responses were confirmed by pressing the space bar. Then, a 500 ms inter‐trial interval preceded the next trial. There were 12 practice trials prior to the formal task. The WM task comprised 80 trials, with each model pair presented four times and each individual model serving as the adjustment target twice. The task duration was ~20 min. Immediately following the WM task, participants completed a surprise LTM task (Figure 1C). Participants viewed 20 model pairs, of which 60% were identical to previously presented pairs and 40% consisted of recombined models from different paired sources. Participants indicated whether each pair had been presented previously by pressing designated response keys. This task lasted approximately 5 min.

### 2.4. Statistical Analysis

Statistical analyses were conducted by JASP (Version 0.18.3.0). For the VS task in Experiment 1, accuracy rates approached ceiling levels in both groups (>97%), so that reaction time (RT) was used as the primary outcome measure. Trials with incorrect responses and trials with RTs exceeding ±2.5 standard deviations from each participant’s condition‐specific mean were excluded from analysis. A mixed‐design ANOVA was conducted with group (MDD vs. HCS) as the between‐subjects factor and condition (facing vs. non‐facing) as the within‐subjects factor.

For the WM task in Experiment 2, spatial error was defined as the numerical difference between the reproduced inter‐model distance and the original distance, measured in pixels. Negative values indicated distance underestimation, whereas positive values indicated distance overestimation. The use of signed spatial error, rather than absolute error, as the primary measure was consistent with previous studies using this spatial WM task [6, 35, 51]. By design, overestimation and underestimation were inclined to occur in the non‐facing and facing conditions, respectively, so that larger between‐condition effects (i.e., non‐facing errors–facing errors) would index strengthened social binding ability [6]. Trials with spatial errors exceeding ±2.5 standard deviations from the condition‐specific mean were excluded. For the LTM task, recollection accuracy served as the dependent measure. Mixed‐design ANOVAs were conducted separately for the two memory tasks using the same factorial structure as in Experiment 1.

To examine associations between task performance and clinical symptoms, Pearson correlation coefficients (*r*) were calculated between symptom severity scores and between‐condition difference effects derived from each task. According to the Shapiro–Wilk test, the depressive symptom scales, including BDI and HAMD, did not follow a normal distribution (ps <0.01 in both MDD samples), so the corresponding Spearman correlation coefficients (rho) were further calculated for these two scales. All other symptom scales used for correlation analyses followed a normal distribution. To ensure the convenience for interpretation, behavioral effects were individually defined for each task. For Experiment 1, the VS effects included ACC (facing–non‐facing) and RT (non‐facing–facing) measures. For Experiment 2, the WM effects included the signed error measure (non‐facing–facing), and the LTM effects included ACC, d‐prime, and criterion *C* measures (all were calculated by facing–non‐facing).

## 3. Results

### 3.1. Experiment 1

For Experiment 1, the data trimming procedure excluded 4.13% trials for HCS and 3.85% trials for patients with MDD. Statistics of all measures are reported in Table 2. The accuracy in both HCS (facing: 98.5%; non‐facing: 96.9%) and MDD (facing: 98.1%; non‐facing: 97.4%) was close to ceiling. There was no significant between‐group difference in overall accuracy (HCS: 97.7% vs. MDD: 97.8%, *F* (1,70) = 0.898, *p* = 0.898), nor were there significant interaction effects between condition and group (*F*(1,70) = 1.335, *p* = 0.252). Therefore, we emphasized the RT analyses.

**Table 2:** Statistics of all measures in three experiment tasks.

Measures	HCS		MDD	
	Facing condition	Non‐facing condition	Facing condition	Non‐facing condition
Exp.1: Visual search task				
Accuracy	0.985 (0.021)	0.969 (0.030)	0.981 (0.031)	0.974 (0.034)
RT (s)	1.092 (0.203)	1.233 (0.250)	1.525 (0.356)	1.794 (0.531)
Log‐transformed RT	0.073 (0.174)	0.191 (0.196)	0.393 (0.252)	0.541 (0.301)
Exp.2: WM task				
Signed error (pixel)	−7.809 (20.10)	−3.243 (18.61)	0.977 (15.38)	1.402 (17.00)
Absolute error (pixel)	34.86 (13.553)	33.43 (13.436)	30.30 (8.964)	30.72 (9.133)
RT (s)	4.044 (1.621)	3.924 (1.461)	6.874 (1.808)	6.626 (1.821)
Log‐transformed RT	1.281 (0.369)	1.260 (0.348)	1.852 (0.252)	1.816 (0.252)
Exp.3: LTM task				
Accuracy	0.592 (0.152)	0.503 (0.176)	0.475 (0.178)	0.525 (0.132)
Hit rate	0.664 (0.202)	0.495 (0.227)	0.521 (0.229)	0.452 (0.201)
False‐alarm rate	0.528 (0.280)	0.486 (0.240)	0.593 (0.226)	0.377 (0.222)
*d*‐prime	0.424 (0.937)	0.028 (1.032)	−0.226 (1.073)	0.257 (0.771)
*C*	−0.287 (0.610)	0.023 (0.503)	−0.197 (0.470)	0.253 (0.504)

*Note:* Log‐transformed RT indicates the natural logarithm (ln) of the raw RT. Data are reported by mean (s.d.).

Abbreviations: HCS, healthy control subjects; LTM, long‐term memory; MDD, major depressive disorder; RT, response time; WM, working memory.

**Figure 2:**
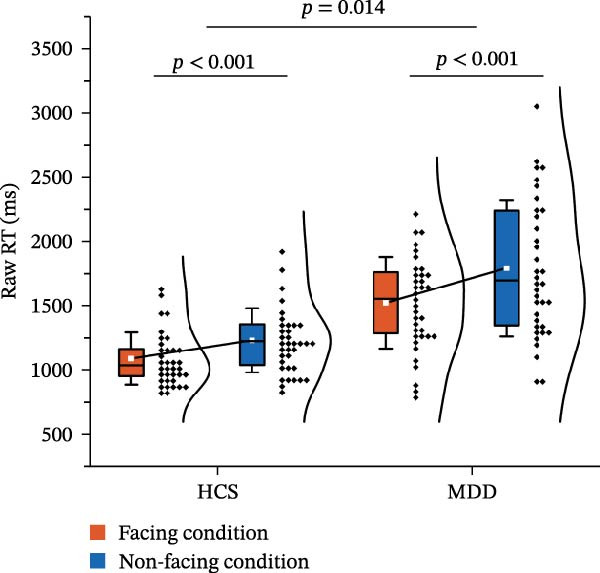
Response time (RT) in Experiment 1. Box‐whisker plot with mean (in‐box white square), median (in‐box black line), 25% and 75% percentiles, and standard deviation (whiskers) across conditions and groups (HCS: healthy control subjects; MDD: major depressive disorder). Individual statistics are displayed by black diamonds, with kernal smoothed distributions. Orange and blue colors represent the facing and non‐facing conditions, respectively.

In general, one‐way ANOVA revealed an overall significantly longer RT for MDD patients than HCS (1.66 s vs. 1.16 s, *F* (1,70) = 38.201, *p* < 0.001, *η_p_* ^2^ = 0.353), and one‐way repeated‐measures ANOVA indicated that VS RT in the facing condition was significantly shorter than in the non‐facing condition (1.31 s vs. 1.51 s, *F* (1,71) = 60.842, *p* < 0.001, *η_p_* ^2^ = 0.461). To compare the cross‐group discrepancy in VS efficiency between the two conditions, our two‐way mixed ANOVA analysis identified significant interaction effects between group and condition (*F*(1,70) = 6.302, *p* = 0.014, *η_p_* ^2^ = 0.083), suggesting an even larger raw RT difference between the facing and non‐facing conditions in MDD (mean difference (MD) = 0.27 s, SD = 0.28) than HCS (MD = 0.14 s, SD = 0.12). As illustrated in Figure 2, post‐hoc analyses showed that the search speed for facing dyads was significantly faster than for non‐facing dyads in both MDD (1.53 s vs. 1.79 s, *t* (70) = −7.493, *p* < 0.001, Cohen’s *d* = −0.752) and HCS (1.09 s vs. 1.23 s, *t* (70) = −3.943, *p* < 0.001, Cohen’s *d* = −0.396) groups. As such, MDD patients preserved attentional advantages for social interaction information as HCS during the VS.

To rule out possible confounding effects of baseline psychomotor slowing or scaling effects, analyses were further conducted using log‐transformed RT. The main effects of group (*F*(1,70) = 38.76, *p* < 0.001, *η_p_* ^2^ = 0.356) and condition (*F*(1,70) = 95.579, *p* < 0.001, *η_p_* ^2^ = 0.577) remained significant, but the group‐by‐condition interaction effect became non‐significant (*F*(1,70) = 1.221, *p* = 0.273, *η_p_* ^2^ = 0.017). Post‐hoc analyses showed that the VS RT was still significantly shorter for the facing condition than the non‐facing condition in both MDD (mean difference (MD) = −0.148, SE = 0.019; *t* (70) = −7.694, *p* < 0.001, Cohen’s *d* = −0.629) and HCS (MD = −0.118, SE = 0.019; *t* (70) = −6.132, *p* < 0.001, Cohen’s *d* = −0.502) groups. As such, patients with MDD preserved the benefits of VS efficiency for socially interactive stimuli, with a comparable magnitude to that of the HCS.

### 3.2. Experiment 2

**Figure 3:**
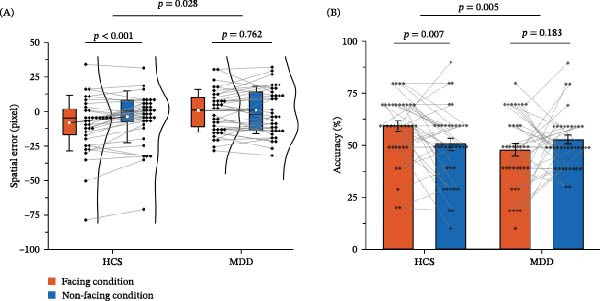
Results across conditions and groups (HCS: healthy control subjects; MDD: major depressive disorder) in Experiment 2. (A) Spatial error (in pixels) for the spatial working memory task. (B) Recall accuracy (%) for the long‐term memory task. Individual statistics are displayed by diamonds, and between‐condition repeatedly measured data are connected with gray lines. Orange and blue colors represent the facing and non‐facing conditions, respectively.

For the WM task in Experiment 2, the data trimming procedure excluded 1.25% trials for HCS and 1.25% trials for MDD patients. Statistics of all measures are reported in Table 2. There were no significant group main effects (HCS vs. MDD: −5.529 vs. 1.190 pixels, *F* (1,70) = 2.679, *p* = 0.106, *η_p_* ^2^ = 0.037). As for the main effect of condition, there was a significant difference in memory errors between the two conditions (facing vs. non‐facing: −3.416 vs. −0.921 pixels, *F* (1,71) = 6.942, *p* = 0.010, *η_p_* ^2^ = 0.089), indicating that socially interactive dyads were memorized as being closer in WM than noninteractive dyads in general. According to the two‐way mixed ANOVA analysis, we identified a significant interaction effect between group and condition (*F*(1,70) = 5.052, *p* = 0.028, *η_p_* ^2^ = 0.067), suggesting that the intimacy effect in WM caused by social interaction was significantly different between the MDD and HCS groups. As illustrated in Figure 3A, post‐hoc analyses showed that only HCS memorized facing dyads significantly closer than non‐facing dyads (−7.809 vs. −3.243 pixels, *F* (1,35) = 14.389, *p* < 0.001, *η_p_* ^2^ = 0.291), while MDD patients (0.977 vs. 1.402 pixels, *F* (1,35) = 0.093, *p* = 0.762, *η_p_* ^2^ = 0.003) did not memorize them differently. Hence, only HCS displayed an advantage for social interaction information in narrowing interpersonal distance in WM, but MDD patients did not show such a spatial compression phenomenon facilitated by social intimacy.

To exclude alternative explanations, additional analyses on absolute spatial errors and RT were conducted. There were no significant group (MDD vs. HCS = 30.51 vs. 34.16 pixels; *F* (1,70) = 1.958, *p* = 0.166, *η_p_* ^2^ = 0.027) or group‐by‐condition (*F*(1,70) = 1.495, *p* = 0.226, *η_p_* ^2^ = 0.021) effects, indicating that the accuracy of WM was comparable between the two groups. Hence, the between‐group differences in the spatial distance compression effect were likely induced by the social attribute, rather than impaired WM capability. Comparisons between all spatial error measures and zero for both groups are provided in Supporting Information 1: Table S1. For this self‐paced WM task, the group‐by‐condition interaction effects of RT were not significant (raw RT: *F* (1,70) = 1.516, *p* = 0.222, *η_p_* ^2^ = 0.021; log‐transformed RT: *F* (1,70) = 1.075, *p* = 0.303, *η_p_* ^2^ = 0.015). Hence, any possible RT difference caused by social interaction in this WM task was similar between the two groups, ruling out potentially different influences of motor ability between the two conditions across the HCS and MDD groups.

As for the LTM task in Experiment 2, there were neither significant group main effects (HCS vs. MDD: 54.7% vs. 50.0%, *F* (1,70) = 2.627, *p* = 0.110, *η_p_* ^2^ = 0.036), nor significant effects of condition (facing vs. non‐facing: 53.3% vs. 51.4%, *F* (1,71) = 0.587, *p* = 0.446, *η_p_* ^2^ = 0.008). However, there was a significant interaction effect between group and condition (*F*(1,70) = 8.252, *p* = 0.005, *η_p_* ^2^ = 0.105), suggesting that the discrepancy in LTM traces between the two conditions was dissimilar between the two participant groups. As illustrated in Figure 3B, post‐hoc analyses showed that only HCS recalled the pairing information of facing dyads significantly better than that of non‐facing dyads (59.2% vs. 50.3%, *F* (1,35) = 8.058, *p* = 0.007, *η_p_* ^2^ = 0.187), while MDD patients did not show much difference in recall accuracy between these two conditions (47.5% vs. 52.5%, *F* (1,35) = 1.842, *p* = 0.183, *η_p_* ^2^ = 0.050). In this way, only HCS manifested an advantage for social interaction information in strengthening LTM traces, whereas MDD patients did not show such facilitation.

Since the overall performance of the LTM task appeared close to chance level, metrics representing detection sensitivity (*d*‐prime) and response criterion (*C*) were further analyzed based on the hit rates and false‐alarm rates. Crucially, the group‐by‐condition interaction effect was significant for *d*‐prime (*F*(1,70) = 9.102, *p* = 0.004, *η_p_* ^2^ = 0.115), but not significant for *C* (*F*(1,70) = 0.772, *p* = 0.383, *η_p_* ^2^ = 0.011). Furthermore, breaking down the performance revealed that the interaction effect was significant for the false alarm rate (*F*(1,70) = 6.151, *p* = 0.016, *η_p_* ^2^ = 0.081), but not for the hit rate (*F*(1,70) = 2.204, *p* = 0.142, *η_p_* ^2^ = 0.031). These results indicated that the observed interaction in ACC was driven by differences in LTM sensitivity rather than mere shifts in response bias.

### 3.3. Symptomatic Correlations

There were no significant correlations between symptom measures and any attention or memory effects elicited by socially interactive information (Supporting Information 1: Table S2). This suggested that alterations in social cognition regarding social interaction in MDD, especially regarding its influences on WM and LTM, might be independent of the current depressive severity.

## 4. Discussion

The present study was intended to investigate whether individuals with a major depressive condition exhibit cognitive advantages for socially interactive cues in the attention and memory domains (Supporting Information 2: STROBE checklist). We found that MDD patients preserved an attentional advantage, reflected by their significantly faster searching speed for facing dyads than non‐facing dyads, but they did not show any advantages in WM or LTM, as the facing dyads were not memorized as spatially closer or recalled with higher accuracy. There were no significant correlations between the three observed effects and clinical symptoms. From a novel perspective of allocentric social interaction processing, our findings underscore the differential cognitive profiles of MDD in different domains, which could be conducive to designing targeted clinical interventions and improving the functional outcomes of depressive individuals.

To our knowledge, this is the first study specifically investigating social interaction processing in MDD from an allocentric perspective. At present, little is known about how MDD patients attend to socially interactive cues and how their memory is modulated by such stimuli. In healthy individuals, interactive face‐to‐face stimuli introduce a few advantages in cognitive processing, such as increased attentional sensitivity, expedited visual discrimination, and enhanced memory traces, which are not recruited by the noninteractive back‐to‐back stimuli [6, 9, 51–53]. The current study found that social interaction could effectively modulate attention allocation in MDD just as in HCS, but could not engender typical advantageous effects in either WM or LTM. As such, the processing of social interactions seemed to show differential patterns across different cognitive domains in MDD.

Crucially, translating these cognitive findings to real‐world behavioral symptoms helps clarify the clinical phenotypes of MDD. Individuals with greater severity of depressive symptoms show a higher likelihood to engage in smaller dyadic social networks [19] and a decreased likelihood to interact with mutual friends (both individuals nominating each other as friends), which typically involve a higher density of social attributes [18]. Our identified differential pattern of intact attention but impaired memory for social interactions provides a preliminary cognitive mechanism for these phenomena. Although patients with MDD can successfully perceive and attend to social interactions happening around them, they do not prioritize and continuously retain this relational map in memory. According to extant theoretical frameworks, MDD is characterized by a hyperactive nonreward system [23] and negatively biased social expectations [22]. Consequently, patients may experience the attended social environment as overwhelming and unrewarding, driving them to actively choose smaller interactive social groups, exhibit decreased motivation for interacting with mutual friends, and maintain an extended comfortable interpersonal distance [54].

Contrary to our hypothesis, the social attention of MDD patients was retained in the current VS task (Experiment 1), with a comparable magnitude of VS advantage for socially interactive stimuli as HCS. Current findings suggest a retained integration of social elements as one social unit in MDD during cognitive perception [5, 55], so that their searching efficiency for socially interactive stimuli was improved. Previous studies using the same task in clinical populations with other neuropsychiatric conditions have similarly reported preservation of social attention for socially interactive information, such as schizophrenia patients [34] and autistic adults [36]. Our findings have extended this effect to MDD, and provided supportive evidence for this cognitive profile, which also corroborates existing findings about comparable social attention during VS in MDD [56–59]. In fact, these populations have been characterized by atypical attention functioning in different nonsocial cognitive domains [60–64], but they have consistently shown attention advantages for social interaction information. One possibility is that an extra social relation was introduced in the facing condition, which was additional to the simple composition of single model elements, and such social binding effects [7–9] were superimposed on the basic attentional alterations, leading to the supposed social advantages during VS. A few studies argued that VS advantages for social interaction might primarily result from attentional cueing effects as a by‐product of element integration [65–67]. However, considering that attentional advantages for social interaction were identified in the context of fundamental attentional alterations in MDD and other neuropsychiatric disorders, certain social messages could still be embodied in the current design and further modulate attention allocation during VS.

On the other hand, the memory advantages of social interaction, including compressed interpersonal spatial distance in WM and higher recall accuracy in LTM, only occurred in HCS, but not in MDD patients. The differential patterns of attention and memory profiles of social interaction processing in MDD might be associated with changes in the representation strength of social binding relationships during perception and memory maintenance. It is suggested that attention could be directed to internal memory representations and prolong the binding of integrated units [68], but the maintained representations will still decay over time [69]. It is likely that the binding of socially interactive models decayed quickly during memory maintenance in MDD patients, so that the differences in spatial WM between the facing and non‐facing conditions disappeared. Since the LTM task in this study measured incidental representations of task‐irrelevant pairing information, the subtle differences in memory traces between the two conditions could be even weaker in MDD patients, especially with their lower social expectations and faster decay of social binding traces [22]. In addition, despite the prominent negative emotion bias in the memory of MDD patients [32], recent studies using nonemotional stimuli have identified stronger or more fixed internal processing during memory retention in MDD [70]. As a result, even if prominent alterations in neural oscillatory activities and event‐related potentials were identified in MDD patients during memory maintenance, their recall accuracy in memory tasks was comparably high to that of HCS [71, 72]. In our Experiment 2, the WM spatial error was slightly lower in patients with MDD than HCS with no significant group main effect, which corroborated their comparable internal representations during memory retention. In this case, the memory representations of both facing and non‐facing dyads could have been internally enhanced, thus mitigating the between‐condition differences in memory tasks. Hence, with decayed binding of socially interactive stimuli and strong internal representations for both conditions, the supposed advantage of social interaction did not occur in MDD. Given the possible floor effects in the accuracy measure of the LTM task, caution should be exercised when interpreting relevant findings.

Our correlation analyses identified no significant associations between attention or memory effects driven by social interactions and the measures of depressive symptoms. According to our Experiment 1, the attentional advantage of social interaction was preserved in the MDD group, and its independence from depressive symptom severity was expected. As for the absence of memory advantages, the lack of symptom correlations was consistent with existing literature regarding social cognition under depressive conditions [73, 74]. Previous studies have highlighted that social and cognitive impairments often operate somewhat independently from acute mood symptoms [75], which remain profound in remitted MDD patients [76] and even 20 years from illness onset [77, 78]. Compared to cognitive changes, depressive symptoms have unique pathways to predict functional outcomes in MDD [79] and do not mediate the relationship between social cognitive deficits and psychosocial dysfunction [80]. Therefore, the current findings suggest that the disappearance of the social interaction advantage in memory may represent a categorical state effect or a core illness‐associated feature. In other words, the onset of a clinical depressive episode or exceeding a certain severity threshold may be sufficient to disrupt these social memory processes, independent of continuous fluctuations in acute symptom severity [81]. However, given the cross‐sectional nature of our data, over‐interpretations of these null correlations should be cautioned.

There are a few clinical implications of the current findings. First, methodologically, our study provides firsthand evidence that social cognition in MDD should be evaluated across subordinate cognitive domains rather than as a monolithic construct. Since social attention remains intact while social memory is impaired, traditional explicit social tasks might be less sensitive in detecting these intricate cognitive changes. Our allocentric perspective and particular paradigms could be translated into sensitive and digitally administered behavioral screening tools to track social cognitive changes in MDD. Second, our findings offer a highly specific avenue for targeted clinical interventions. Crucially, since the perceptual binding and attentional capture of social interactions are preserved in MDD, therapeutic efforts specifically targeting the top‐down valuation and memory maintenance of social cues could be promising. For instance, noninvasive brain stimulation combined with specialized computerized memory training could be used to enhance the retention of socially interactive information. Ultimately, improving this specific domain of social memory may help MDD patients reconstruct their relational maps, mitigate interpersonal conflicts, and foster sustainable real‐world social engagement.

There are several limitations to the current study. First, general cognitive ability and social functioning were not assessed in this study. Although the alternative explanations for impaired feature binding or WM abilities could be modestly ruled out by the existence of social attention benefits and the nonsignificant group effects of absolute error values, we still acknowledge that the performance of MDD patients in these social tasks might have been influenced by unmeasured general cognitive impairments. Future studies are encouraged to use nonhuman object controls to systematically disentangle general cognitive influences from social cognition in MDD [82]. Second, illness‐related characteristics could potentially influence attention and memory performance, and whether such influences interact with social cognition requires further examination. Third, patients were tested in a hospital setting and HCS were tested in a university laboratory, which might have introduced an environmental confound. Fourth, the VS task in Experiment 1 exclusively used male dyads to control for confounding higher‐order social semantics. While this isolated the attention effects induced by facingness manipulations, we acknowledge it may limit the broader ecological validity of our findings. Future studies should incorporate diverse sex configurations (e.g., female–female and mixed‐sex dyads) to thoroughly verify the generalizability of this social cognitive performance across genders. Finally, and importantly, social attention and memory were evaluated in separate experiments using different participant samples. Consequently, the differential findings across these cognitive domains demonstrate a between‐sample pattern rather than a true within‐person dissociation. Because each participant completed only one experiment, the current design cannot definitively determine whether the same individuals with depression who exhibit preserved social attention also show impaired social memory. Future research employing within‐subject designs is necessary to confirm whether these contrasting cognitive profiles coexist at the individual level.

## 5. Conclusions

In conclusion, this study revealed different patterns of social interaction advantages in the attention and memory domains of social cognition in individuals with MDD. Compared to the noninteractive cues, patients with MDD exhibited a strengthened attentional advantage for facing dyads during VS, but they did not memorize them as spatially closer in WM or recall them more accurately in LTM, showing alterations in social memory for such information. These findings are noteworthy because they introduce a novel perspective, i.e., allocentric social interaction processing, for the investigation of social cognition in MDD and encourage more microscopic investigations in different cognitive domains.

## Author Contributions

**Enze Tang**: conceptualization, data curation, formal analysis, investigation, methodology, software, visualization, writing – original draft, writing – review and editing. **Xiao-Mei Zhang**: investigation, funding acquisition, project administration, resources, writing – review and editing. **Jian Li:** methodology, software, validation, writing – review and editing. **Yueyao Liu:** funding acquisition, validation, writing – review and editing. **Zhong-Xia Shen:** funding acquisition, project administration, resources, supervision, writing – review and editing. **Hui Chen:** conceptualization, data curation, funding acquisition, project administration, resources, supervision, writing – review and editing.

## Funding

This work was supported by Grants from the Science and Technology Innovation 2030—“Brain Science and Brain‐like Research” Major Project (Grant 2022ZD0210800), the National Natural Science Foundation of China (Grants 32441105 and 32171046), the Fundamental Research Funds for the Central Universities (Grants 226‐2024‐00207 and 226‐2024‐00118), the Zhejiang Key Laboratory of Neurocognitive Development and Mental Health (Grant 2025E10037) awarded to Hui Chen; the Open Research Fund of the Zhejiang Key Laboratory of Precision Psychiatry (Grant 2025B5) awarded to Yueyao Liu; the Huzhou Public Welfare Research Project Social Development Category (Grants 2024GYB14 and 2024GYB31, ZS) awarded to Zhong‐Xia Shen and Xiao‐Mei Zhang; Jiangsu Funding Program for Excellent Postdoctoral Talent, awarded to Jian Li.

## Ethics Statement

All procedures performed in this study were approved by the institution review board at the Department of Psychology and Behavioral Sciences, Zhejiang University (2022087).

## Conflicts of Interest

The authors declare no conflicts of interest.

## Supporting Information

Additional supporting information can be found online in the Supporting Information section.

## Supporting information


**Supporting Information 1** Table S1: Comparisons between spatial error measures and zero in the WM task of Experiment 2. Table S2. Correlations between clinical symptoms and primary outcomes.

**Supporting Information 2** STROBE checklist: Checklist of items that should be included in reports of observational studies.

## Data Availability

The data that support the findings of this study are available from the corresponding author upon reasonable request.
