# Supplementary material for: Differential Patterns of Social Attention and Memory Profiles in Depression: Evidence From Third‐Person Social Interaction Processing
Source: Depress Anxiety. 2026 Jun 30;2026:7518415. doi: 10.1155/da/7518415 (PMC13316134; doi:10.1155/da/7518415)
Supplement: Supplementary file 1 — Supporting Information 1 Table S1: Comparisons between spatial error measures and zero in the WM task of Experiment 2. Table S2. Correlations between clinical symptoms and primary outcomes. [file DA-2026-7518415-s002.docx]

**Supplementary Table 1. Comparisons between spatial error measures and zero in the WM task of Experiment 2**

| **Group** | **Measure** | **Value** | **Statistics** |
| --- | --- | --- | --- |
| HCS |  |  |  |
|  | FF error | -7.809 (20.097) | F(1,35) = 5.435, p = 0.026, η_p_^2^ = 0.134 |
|  | BB error | -3.243 (18.607) | F(1,35) = 1.093, p = 0.303, η_p_^2^ = 0.030 |
|  | Total error | -5.529 (19.016) | F(1,35) = 3.043, p = 0.090, η_p_^2^ = 0.080 |
|  | FF absolute error | 34.863 (13.553) | F(1,35) = 238.2, p < 0.001, η_p_^2^ = 0.872 |
|  | BB absolute error | 33.428 (13.436) | F(1,35) = 222.8, p < 0.001, η_p_^2^ = 0.864 |
|  | Total absolute error | 34.157 (13.008) | F(1,35) = 248.2, p < 0.001, η_p_^2^ = 0.876 |
| MDD |  |  |  |
|  | FF error | 0.977 (15.376) | F(1,35) = 0.145, p = 0.705, η_p_^2^ = 0.004 |
|  | BB error | 1.402 (17.003) | F(1,35) = 0.245, p = 0.624, η_p_^2^= 0.007 |
|  | Total error | 1.190 (15.654) | F(1,35) = 0.208, p = 0.651, η_p_^2^ = 0.006 |
|  | FF absolute error | 30.300 (8.964) | F(1,35) = 411.3, p < 0.001, η_p_^2^ = 0.922 |
|  | BB absolute error | 30.715 (9.133) | F(1,35) = 407.2, p < 0.001, η_p_^2^ = 0.921 |
|  | Total absolute error | 30.514 (8.595) | F(1,35) = 453.7, p < 0.001, η_p_^2^ = 0.928 |

Note. HCS = healthy controls; FF = face-to-face condition; BB = back-to-back condition; MDD = major depressive disorder. Values are reported by mean (s.d.).

**Supplementary Table 2. Correlations between clinical symptoms and primary outcomes**

| Behavior Measures | | PANAS-P | | PANAS-N | | PHQ-9 | | STAI-S | | STAI-T | | BDI-II | | | | HAMD | | | |
| --- | --- | --- | --- | --- | --- | --- | --- | --- | --- | --- | --- | --- | --- | --- | --- | --- | --- | --- | --- |
|  |  | r | p | r | p | r | p | r | p | r | p | r | p | rho | p | r | p | rho | p |
| Exp 1: VS | |  |  |  |  |  |  |  |  |  |  |  |  |  |  |  |  |  |  |
|  | RT effect | -0.022 | 0.900 | -0.011 | 0.951 | -0.175 | 0.308 | -0.058 | 0.736 | -0.077 | 0.656 | -0.156 | 0.362 | -0.196 | 0.253 | 0.014 | 0.937 | -0.021 | 0.904 |
|  | ACC effect | 0.213 | 0.213 | -0.166 | 0.333 | 0.029 | 0.868 | -0.219 | 0.200 | -0.142 | 0.408 | -0.049 | 0.778 | -0.109 | 0.528 | -0.185 | 0.279 | -0.278 | 0.101 |
| Exp 2: WM | |  |  |  |  |  |  |  |  |  |  |  |  |  |  |  |  |  |  |
|  | Signed error | -0.092 | 0.594 | 0.123 | 0.473 | 0.165 | 0.337 | -0.072 | 0.676 | -0.014 | 0.934 | 0.117 | 0.496 | 0.030 | 0.863 | -0.080 | 0.642 | -0.181 | 0.290 |
| Exp 2: LTM | |  |  |  |  |  |  |  |  |  |  |  |  |  |  |  |  |  |  |
|  | ACC | -0.171 | 0.318 | 0.187 | 0.275 | -0.077 | 0.657 | 0.033 | 0.848 | 0.005 | 0.977 | -0.251 | 0.140 | -0.215 | 0.207 | -0.148 | 0.389 | -0.130 | 0.451 |
|  | d-prime | -0.150 | 0.384 | 0.209 | 0.222 | -0.083 | 0.631 | 0.077 | 0.655 | 0.016 | 0.926 | -0.226 | 0.185 | -0.159 | 0.355 | -0.124 | 0.471 | -0.060 | 0.727 |
|  | C | 0.078 | 0.650 | 0.049 | 0.777 | -0.087 | 0.612 | 0.077 | 0.657 | 0.002 | 0.992 | 0.063 | 0.717 | 0.129 | 0.453 | -0.077 | 0.657 | 0.060 | 0.730 |

***Notes.*** VS: visual search; WM: working memory; LTM: long-term memory; PANAS: Positive and Negative Affect Scale (positive or negative subscales); PHQ-9: 9-item Patient Health Questionnaire; STAI: State-Trait Anxiety Inventory (state or trait subscales); BDI-II: Beck Depression Inventory Second Edition; HAMD: Hamilton Depression Scale. Pearson correlation coefficients (r) are reported for scales with a normal distribution, and Spearman correlation coefficients (rho) are additionally reported for scales with a non-normal distribution.
