# Supplementary material for: Differential Patterns of Social Attention and Memory Profiles in Depression: Evidence From Third‐Person Social Interaction Processing
Source: Depress Anxiety. 2026 Jun 30;2026:7518415. doi: 10.1155/da/7518415 (PMC13316134; doi:10.1155/da/7518415)
Supplement: Supplementary file 2 — Supporting Information 2 STROBE checklist: Checklist of items that should be included in reports of observational studies. [file DA-2026-7518415-s001.docx]

STROBE Statement—checklist of items that should be included in reports of observational studies

|  | Item No. | Recommendation | Page  No. | Relevant text from manuscript |
| --- | --- | --- | --- | --- |
| **Title and abstract** | 1 | (*a*) Indicate the study’s design with a commonly used term in the title or the abstract | 2 | “this study employed two cross-sectional experiments” |
|  |  | (*b*) Provide in the abstract an informative and balanced summary of what was done and what was found | 2 | “A total of 72 patients ...... for interactive dyads in long-term memory” |
| Introduction | | | |  |
| Background/rationale | 2 | Explain the scientific background and rationale for the investigation being reported | 3-6 | “Major depressive disorder (MDD) is a severe psychiatric disorder ...... cognitive prioritization for socially interactive stimuli.” |
| Objectives | 3 | State specific objectives, including any prespecified hypotheses | 6 | “The present study aimed to investigate ...... social interaction across attention and memory domains.” |
| Methods | | | |  |
| Study design | 4 | Present key elements of study design early in the paper | 6 | “Experiment 1 assessed attentional processing ...... while minimizing task-irrelevant demands or confounds.” |
| Setting | 5 | Describe the setting, locations, and relevant dates, including periods of recruitment, exposure, follow-up, and data collection | 6 | “Data collection was carried out between July 2024 and April 2025. Participants with MDD were recruited from Huzhou Third Municipal Hospital in China.” |
| Participants | 6 | (*a*) *Cohort study*—Give the eligibility criteria, and the sources and methods of selection of participants. Describe methods of follow-up  *Case-control study*—Give the eligibility criteria, and the sources and methods of case ascertainment and control selection. Give the rationale for the choice of cases and controls  *Cross-sectional study*—Give the eligibility criteria, and the sources and methods of selection of participants | 6-7 | “Inclusion criteria for the MDD group were as follows ...... Written informed consent was obtained from all participants prior to participation.” |
|  |  | (*b*) *Cohort study*—For matched studies, give matching criteria and number of exposed and unexposed  *Case-control study*—For matched studies, give matching criteria and the number of controls per case | NA | NA |
| Variables | 7 | Clearly define all outcomes, exposures, predictors, potential confounders, and effect modifiers. Give diagnostic criteria, if applicable | 10-11 | “Statistical analyses were conducted ...... d-prime and criterion C measures (all were calculated by Facing - Non-facing)” |
| Data sources/ measurement | 8* | For each variable of interest, give sources of data and details of methods of assessment (measurement). Describe comparability of assessment methods if there is more than one group | 8-10 | “Experiments were programmed and executed by MATLAB ...... This task lasted approximately five minutes.” |
| Bias | 9 | Describe any efforts to address potential sources of bias | 8, 10-11 | “For Experiment 1, pictures of two side-view male models ...... against a uniform background depicting an empty room.”  “Trials with incorrect responses and trials ...... the same factorial structure as in Experiment 1” |
| Study size | 10 | Explain how the study size was arrived at | 6 | “Prior power analyses were conducted ...... were recruited for Experiment 1 and Experiment 2, respectively.” |

Continued on next page

| Quantitative variables | 11 | Explain how quantitative variables were handled in the analyses. If applicable, describe which groupings were chosen and why | 10-11 | “Statistical analyses were conducted ...... d-prime and criterion C measures (all were calculated by Facing - Non-facing)” |
| --- | --- | --- | --- | --- |
| Statistical methods | 12 | (*a*) Describe all statistical methods, including those used to control for confounding | 10-11 | “Statistical analyses were conducted ...... d-prime and criterion C measures (all were calculated by Facing - Non-facing)” |
|  |  | (*b*) Describe any methods used to examine subgroups and interactions | 10-11 | “Statistical analyses were conducted ...... d-prime and criterion C measures (all were calculated by Facing - Non-facing)” |
|  |  | (*c*) Explain how missing data were addressed | 10-11 | “Statistical analyses were conducted ...... d-prime and criterion C measures (all were calculated by Facing - Non-facing)” |
|  |  | (*d*) *Cohort study*—If applicable, explain how loss to follow-up was addressed  *Case-control study*—If applicable, explain how matching of cases and controls was addressed  *Cross-sectional study*—If applicable, describe analytical methods taking account of sampling strategy | 10-11 | “Statistical analyses were conducted ...... d-prime and criterion C measures (all were calculated by Facing - Non-facing)” |
|  |  | (*e*) Describe any sensitivity analyses | 10-11 | “Statistical analyses were conducted ...... d-prime and criterion C measures (all were calculated by Facing - Non-facing)” |
| Results | | | | |
| Participants | 13* | (a) Report numbers of individuals at each stage of study—eg numbers potentially eligible, examined for eligibility, confirmed eligible, included in the study, completing follow-up, and analysed | 6 | “Therefore, independent samples of 36 patients with MDD and 36 healthy control subjects (HCS) were recruited for Experiment 1 and Experiment 2, respectively.” |
|  |  | (b) Give reasons for non-participation at each stage | NA | NA (cross-sectional, no drop-out) |
|  |  | (c) Consider use of a flow diagram | NA | NA (cross-sectional, no drop-out) |
| Descriptive data | 14* | (a) Give characteristics of study participants (eg demographic, clinical, social) and information on exposures and potential confounders | Table 1 | Table 1 |
|  |  | (b) Indicate number of participants with missing data for each variable of interest | 11-12 | “For Experiment 1, the data trimming procedure excluded 4.13% trials for HCS and 3.85% trials for patients with MDD.”  “For the WM task in Experiment 2, the data trimming procedure excluded 1.25% trials for HCS and 1.25% trials for MDD patients.” |
|  |  | (c) *Cohort study*—Summarise follow-up time (eg, average and total amount) | NA | NA |
| Outcome data | 15* | *Cohort study*—Report numbers of outcome events or summary measures over time | NA | NA |
|  |  | *Case-control study—*Report numbers in each exposure category, or summary measures of exposure | NA | NA |
|  |  | *Cross-sectional study—*Report numbers of outcome events or summary measures | Table 2 | Table 2 |
| Main results | 16 | (*a*) Give unadjusted estimates and, if applicable, confounder-adjusted estimates and their precision (eg, 95% confidence interval). Make clear which confounders were adjusted for and why they were included | 11-14 | “For Experiment 1, the data trimming procedure ...... might be independent of the current depressive severity.” |
|  |  | (*b*) Report category boundaries when continuous variables were categorized | NA | NA |
|  |  | (*c*) If relevant, consider translating estimates of relative risk into absolute risk for a meaningful time period | NA | NA |

Continued on next page

| Other analyses | 17 | Report other analyses done—eg analyses of subgroups and interactions, and sensitivity analyses | 11-14 | “To rule out possible confounding effects of baseline psychomotor slowing or scaling effects ... with a comparable magnitude to that of the HCS.”  “To exclude alternative explanations, additional analyses ...... between the two conditions across the HCS and MDD groups.”  “Since the overall performance of the LTM task appeared ...... rather than mere shifts in response bias.” |
| --- | --- | --- | --- | --- |
| Discussion | | | | |
| Key results | 18 | Summarise key results with reference to study objectives | 14-15 | “The present study was intended to investigate ...... improving the functional outcomes of depressive individuals.” |
| Limitations | 19 | Discuss limitations of the study, taking into account sources of potential bias or imprecision. Discuss both direction and magnitude of any potential bias | 18-19 | “There are several limitations to the current study. ...... whether these contrasting cognitive profiles coexist at the individual level.” |
| Interpretation | 20 | Give a cautious overall interpretation of results considering objectives, limitations, multiplicity of analyses, results from similar studies, and other relevant evidence | 15-19 | “To our knowledge, this is the first study specifically investigating ...... whether these contrasting cognitive profiles coexist at the individual level.” |
| Generalisability | 21 | Discuss the generalisability (external validity) of the study results | 15-18 | “Crucially, translating these cognitive findings to real-world behavioral symptoms ...... and foster sustainable real-world social engagement.” |
| Other information | |  | | |
| Funding | 22 | Give the source of funding and the role of the funders for the present study and, if applicable, for the original study on which the present article is based | 20 | Funding statement. |

*Give information separately for cases and controls in case-control studies and, if applicable, for exposed and unexposed groups in cohort and cross-sectional studies.

**Note:** An Explanation and Elaboration article discusses each checklist item and gives methodological background and published examples of transparent reporting. The STROBE checklist is best used in conjunction with this article (freely available on the Web sites of PLoS Medicine at http://www.plosmedicine.org/, Annals of Internal Medicine at http://www.annals.org/, and Epidemiology at http://www.epidem.com/). Information on the STROBE Initiative is available at www.strobe-statement.org.
